# The cultural origin of saving behavior

**DOI:** 10.1371/journal.pone.0202290

**Published:** 2018-09-12

**Authors:** Joan Costa-Font, Paola Giuliano, Berkay Ozcan

**Affiliations:** 1 Department of Health Policy, London School of Economics, London, United Kingdom; 2 Anderson School of Management, UCLA, Los Angeles, California, United States of America; 3 National Bureau of Economic Research (NBER), Cambridge, Massachusetts, United States of America; 4 Department of Social Policy, London School of Economics, London, United Kingdom; University of Toronto, Rotman School, CANADA

## Abstract

Traditional economic interpretations have not been successful in explaining differences in saving rates across countries. One hypothesis is that savings respond to cultural specific social norms. The accepted view in economics so far is that culture does not have any effect on savings. We revisit this evidence using a novel dataset, which allows us to study the saving behavior of up to three generations of immigrants in the United Kingdom. Against the backdrop of existing evidence, we find that cultural preferences are an important explanation for cross-country differences in saving behavior, and their relevance persists up to three generations.

## Introduction

Savings are important drivers of economic growth and are pre-requisite to the sustainability of pension systems and the international balance of trade. The correlates of differences in saving rates have been well-studied in the literature and include the effect of demographics, differences in income and growth rates, social security systems, tax systems and housing price differentials, and financial markets and liberalization [1, 2, 3, 4, 5, 6, 7]. However, even after controlling for differences in these covariates, a large part of the differences in saving rates across societies remain unexplained [8].

One hypothesis is that savings respond to cultural specific norms. Previous evidence on the relevance of culture for saving behavior comes from [9]. The authors used data from the *Canadian Surveys of Family Expenditures*. They studied the saving behavior of first-generation immigrants in Canada and test whether saving rates varied systematically by place of origin. All immigrants face the same institutional and economic environment, the one of Canada, therefore any systematic difference across places of origin, if any, could be attributed to culture. The authors found that saving patterns did not vary systematically by place of origin. These findings formed the basis of the commonly accepted view that culture does not influence saving behavior.

The original study [9] had various limitations: first, the sample of immigrants was small, second the classification of the place of origin was too broad (the authors did not have any information about the country of origin but only about broad geographical regions), and third wealth was not measured properly.

In this paper, we re-examine the hypothesis that culture matters for saving behavior, by looking at the saving behavior of three generations of immigrants in the United Kingdom and using data from the Understanding Society Survey, the largest UK household longitudinal survey.

Like in the original paper [9], the identification strategy relies on the possibility to observe immigrants from different countries of origin in the same environment (in this case, the United Kingdom). This allows distinguishing cultural determinants of savings from factors like tax code, social security system and any other institutional and economic factor more generally.

The Understanding Society Survey presents numerous advantages. First, it allows us to identify first, second (also defined children of immigrants: individuals born in the UK with parents born abroad) and third generation immigrants (i.e. individuals born in the UK, with parents born in the UK, but with both grand-parents born abroad). The original study [9] had information only on first-generation immigrants, for whom problems of selection and disruption due to immigration are a serious concern. While first-generation immigrants in the UK could also experience the same issues of selection and disruption due to immigration, for second and third generations these concerns should be more limited. However, there is still a potential concern since second and even third-generation immigrants could be discriminated against or might feel conflicted between the UK and their parental country of origin.

Second, the UK is one of the largest immigrant-receiving states with a large variation in the country of origin of up to three generations of immigrants.

Third, the dataset contains detailed information on both actual and self-reported savings for a large sample of immigrants, their children and their grandchildren from different countries of origin.

Various contributions to the economics literature have studied the behavior of immigrants to show the relevance of cultural values for different economic outcomes, such as living arrangements [10], female labor force participation and fertility [11], trust [12] and preferences for redistribution [13]. For a review on the relevance of culture on economic outcomes, see [14] and [15]. All these contributions study the persistence of cultural traits for first or second-generation immigrants. None of the studies mentioned above has been able to go beyond the analysis of second-generation immigrants, as the datasets used did not contain any information on the country of origin of one individual’s grandparents. An exception is the study by [16] that goes beyond second-generation immigrants and looks at the behavior of first, second and ‘higher generation immigrants’ (higher generation in their context are third, fourth or further generation) in the United States using the General Social Survey. Importantly, the authors report evidence of persistence and evolution of different types of values and show that some cultural traits, such as family and gender values, political views, and deep personal religious values, are very persistent. Other traits, instead (such as attitudes towards cooperation, children’s independence, and attitudes towards sexuality) tend to exhibit less persistence. The authors do not look at saving behavior in their analysis.

To study the relevance of cultural norms we link each immigrant to the saving rates from their country of origin. We use a measure of savings rate over GDP, calculated from 1990 until 2010, as a proxy for culture. We attribute the association found in our data between the behaviour of immigrants and the saving rate in the country of origin to differences in cultural beliefs across immigrant groups. Looking at various generations of immigrants constitutes a sort of “natural experiment”. When migrants move to a new country, they leave behind the economic and institutional conditions that determine their saving behavior in the country of origin, they however bring with them their cultural beliefs. Savings/GDP at the aggregate level will depend on the distribution of beliefs about the importance of savings, and this distribution varies across countries, hence reflecting variation in culture. If this aggregate variable has then explanatory power for the variation in immigrants’ saving outcomes, even after controlling for their individual economic attributes, only the cultural component of this variable can be responsible for this correlation. This is because the economic and institutional environment is now the same for immigrants from different countries and it is the one of the United Kingdom. The mechanisms behind the correlation of saving behavior of different generations and saving outcomes in the country of origin can be attributed to intergenerational cultural transmission, according to which parents tend to transmit their beliefs to their children (see [17]). Nonetheless, the measure of savings/GDP from the country of origin proxies an average effect of cultural transmission. A substantial heterogeneity can be at play for different immigrant groups, for example the frequency of contacts with the country of origin or the size of the community of immigrants in the neighbourhood where immigrants live. Unfortunately, the Understanding Society Survey does not contain any information on any of these variables, and we cannot study in this paper this type of heterogeneity.

## Material and methods

### Variable construction and definition

Our main sample consists of individuals older than thirteen. We define the first generation as immigrants who are not born in the UK; second generation as those who are born in the UK but with at least one parent not born in the UK; and third generation, as those who are born in the UK to parents both of whom are also born in the UK, but have at least one grandparent, who is not born in the UK. This is the formal definition of immigrant generations given in the Understanding Society Survey dataset. Natives are those with all grandparents born in the UK. The number of observations for each country of origin is provided in the S1 Table of the Supporting Information.

#### Proxy for culture

Our proxy for culture, savings/GDP of the country of origin, is taken from the World Bank’s *World Development Indicators*. Gross domestic savings are calculated as GDP less final consumption expenditures (i.e. total consumption). We pooled data over the 1990–2010 period to minimize measurement error. The results are however robust to different time ranges (for example if we consider the 1990–1999 and 2000–2010 periods separately). Saving rates across countries for our sample of interest are also highly correlated in the two periods (0.87) and have very similar means (20.3 and 20.7) and standard deviations (7 and 9.04).

#### Outcome variables

We use three measures of saving behavior.

1. *Total amount of saving*: This variable is the self-reported “monthly amount of savings”. This variable is available in the Understanding Society Survey dataset for the respondents who answers “yes” to the “propensity to save” question reported below. These people were asked another follow-up question in wave 2 and wave 4 to collect self-reported data on the monthly amount of savings: “About how much on average do you personally manage to save a month?”. We take the log of this variable and, in order not to lose observations for individuals reporting zero, we added one pound to the total amount reported.
2. *Propensity to save*: This is a self reported binary measure of saving behavior, where 1 indicates that an individual answers “yes” to the following survey question in wave 2 and wave 4. “Do you save any amount of your income, for example by putting something away now and then in a bank, building society, or Post Office account, other than to meet regular bills? Please include share purchase schemes, ISA's and Tessa accounts.” The person can answer either yes or no.
3. *Positive Savings*: We constructed an objective measure of actual savings by using a large set of wealth variables in the wealth module of the survey in waves 2 and 4. Net worth is defined as the sum of housing equity, car equity and liquid financial net worth. We then calculated change in “net worth” from wave 2 to wave 4 as [Net worth(w4)−Net worth_(w2)_]/ 10000. Our variable of interest, “*positive savings”*, takes the value of the change if the wealth has increased between two time periods or zero if wealth has decreased or stayed the same over the same period.

#### Control variables

Our control variables include age dummies, dummy indicators for female and for whether the person is married, number of children, log of monthly total household net income, which is a derived variable available in the dataset (This variable can take negative values, and it is top-coded +/- £20,000. We added 13771.8 + 1 to all observations before taking the log because -13771.8 is the lowest value. This adjustment is done so that we do not loose observations when logs are taken). We also control for employment status (we include dummies for individuals who are unemployed and out of the labor force, the excluded group are the employed), education (we include dummies for secondary education, A-level degree, other higher degree, and college and above; the excluded group includes individuals with the lowest level of education, “no qualification” or “other qualification”), paternal education (we include dummies for whether the father left school with no qualification, whether the father had some qualification, post-school qualification, or college or more; the excluded group are fathers who did not go to school) and occupation fixed effects. We also test the robustness of our results to the inclusion of a measure of permanent income (S3 Table). This measure is calculated using a procedure similar to [18], [19] and, to a certain extent, [20]: we regress our measure of log net household income on all individual characteristics, such as flexible age dummies, gender, marital status, number of children, education, occupational class, region dummies and wave dummies. The predicted income using this regression is used as a measure of individual-specific permanent income (i.e. residuals would constitute the transitory income).

### Descriptive analysis

**Fig 1:**
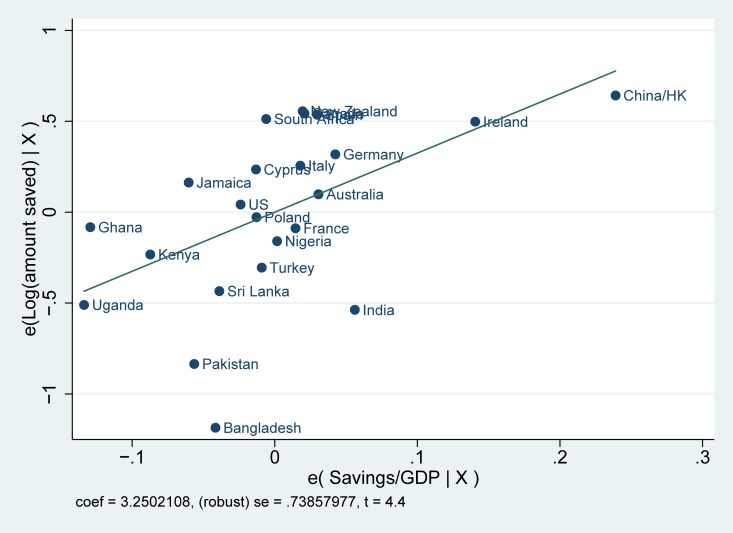
Partial correlation plot: Log (amount saved) for first generation immigrants. Log (amount saved) for first generation immigrants is the logarithm of the self-reported monthly amount of saving. The saving rate in the countries of origin indicates the average gross domestic savings over GDP from 1990–2010.

**Fig 2:**
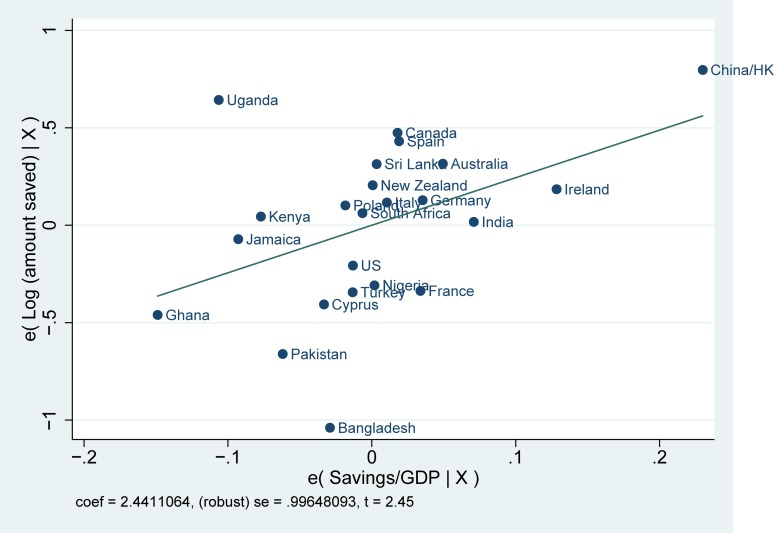
Partial correlation plot: Log (amount saved) for second generation immigrants. Log (amount saved) for second generation immigrants is the log of the self-reported monthly amount of saving divided by the net monthly household income. The saving rate in the countries of origin indicates the average gross domestic savings over GDP from 1990–2010.

**Fig 3:**
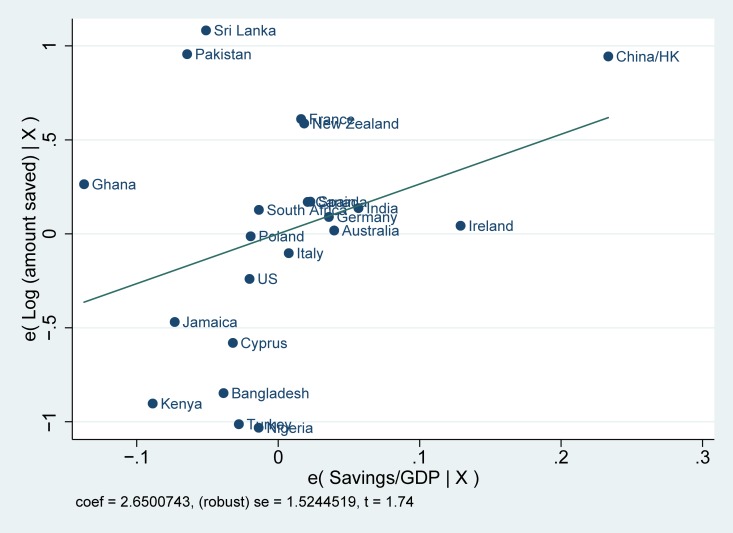
Partial correlation plot: Log (amount saved) for third generation immigrants. Log (amount saved) for third generation immigrants is the log of the self-reported monthly amount of saving divided by the net monthly household income. The saving rate in the countries of origin indicates the average gross domestic savings over GDP from 1990–2010.

Before starting our empirical analysis, we first examine whether there exists a systematic correlation between saving rates in the country of origin and saving behavior among the three generations of immigrants. We report the correlations for the logarithm of the total amount of savings among immigrants and saving rates in the countries of origin in Figs 1–3. A number of facts are apparent from them. First, saving rates are strongly correlated with savings in the country of origin: coming from cultures with high saving rates is reflected in higher saving rates among immigrants in the United Kingdom. Second, these correlations are strong, not only for immigrants and their children, but also for third-generation immigrants. Finally, Figs 1–3 also show that the relationship is not driven by a small number of countries.

### Empirical analysis

The differences in saving rates among immigrants shown in Figs 1–3 could be driven by individual characteristics or family background characteristics or by particular economic conditions of the place where migrants decide to live. We, therefore, turn to OLS multivariate regression estimates of the relationship between saving behavior among immigrants and saving in the country of origin. Using a multivariate regression framework allows us to account for a host of other factors that may also affect savings. Without appropriate controls, we run the risk of capturing a spurious correlation between the unobserved factors and saving in the country of origin.

Our empirical analysis takes care of these concerns by estimating the following equation:

$$Y_{ic}=\alpha ({Savings/GDP)}_{c}+\beta X_{i}+\theta X_{it}{+\delta }_{t}+\mu_{r}+\mu_{r}∙\delta_{t}+\varepsilon_{ict}$$

where *Y*_*ic*_ are our outcomes of interest (the logarithm of the reported amount of savings, a dummy indicating whether the individual is saving or not, and savings calculated using differences in wealth). *X*_*i*_ and *X*_*it*_ are time invariant and time variant individual controls described above. Our specification also includes a full set of wave and regional dummies (*δ*_*t*_ and *μ*_*r*_). These are the baseline controls used in the first three columns of each table. From columns 4–6 we also add all the non-linear interactions between region and wave fixed effects (*μ*_*r*_ ∙ *δ*_*t*_) to control for regional specific trends that could be driving differences in savings as a result of differences in economic conditions. In this specification, we also include a full set of dummies for the education of the father, described above. The standard errors are clustered at the country of origin level. Descriptive statistics are provided in the S2 Table.

## Results and discussion

The estimates for saving rates are reported in Table 1. From the regression estimates, we see the same pattern emerging as in Figs 1–3. Immigrants coming from countries with high saving rates also tend to save more in the United Kingdom. In addition, the importance of culture plays a role up until the third generation. The coefficients are not only statistically significant, but they are also meaningful in magnitude. Based upon the estimates from column 1, a one standard deviation change in the country of origin savings rate is associated with an increase of saving rates of .051 standard deviations in the first generation, .040 standard deviation in the second generation and .025 standard deviation in the third. The impact seems to be declining across generations. The effect is also meaningful when compared to other economic factors such as the level of education: for the first generation, the effect of savings in the country of origin is equal to forty-one percent of the effect of having a college degree (for which the beta coefficient is equal to 0.104) and fifty-one percent of the effect of income (for which the beta coefficient is equal to 0.124). The effect of culture in the regressions with the extended set of controls (columns 4–6) shows a similar pattern, but with almost no decline from the first to the second generation. The effect on the third generation also loses significance, although the size of the coefficient remains of similar magnitude.

**Table 1:** 'Log-amount saved' self-reported amount of (positive) savings.

Variables	1^st^ Gen	2^nd^ Gen	3^rd^ Gen	1^st^ Gen (b)	2^nd^ Gen (b)	3^rd^ Gen (b)
**Dom. savings/GDP**	1.520**	1.184**	0.849*	1.634**	1.665***	**0.772**
	**(2.782)**	**(2.287)**	**(1.796)**	**(2.797)**	**(2.914)**	**(1.584)**
Female	0.018	-0.049	-0.069	0.001	-0.051	-0.062
	(0.225)	(0.816)	(0.833)	(0.007)	(0.866)	(0.892)
Married	-0.162*	0.001	0.282*	-0.159	0.007	0.265*
	(1.836)	(0.008)	(1.847)	(1.518)	(0.074)	(1.965)
Number of children	-0.142**	-0.183***	-0.302***	-0.141**	-0.230***	-0.338***
	(2.759)	(2.892)	(7.531)	(2.718)	(3.657)	(7.973)
Log Monthly Income	2.549***	3.113***	1.775*	2.590***	3.775***	1.604
	(4.642)	(5.937)	(1.885)	(3.926)	(6.618)	(1.715)
Education *(Ref*. *No Qualification)*						
College and above	0.500***	0.763***	0.717***	0.537***	0.944***	0.839***
	(3.994)	(6.595)	(5.230)	(3.979)	(7.551)	(6.186)
Other higher degree	0.282**	0.268	0.571***	0.301**	0.544***	0.711***
	(2.204)	(1.503)	(3.549)	(2.571)	(3.036)	(4.567)
A-level degree	0.238***	0.119	0.494***	0.288***	0.180	0.488***
	(2.872)	(0.833)	(5.702)	(2.902)	(1.254)	(4.702)
Secondary education	0.224***	0.129	0.243	0.302***	0.211	0.354*
	(3.039)	(1.159)	(1.535)	(4.130)	(1.371)	(1.793)
Employment Status *(Ref*: *Employed)*						
Unemployed	-0.475*	-0.350*	-0.766**	-0.491*	-0.456*	-0.803**
	(2.051)	(2.014)	(2.340)	(1.882)	(1.935)	(2.252)
Out of Labor Force	-0.305	-0.096	-0.350	-0.244	-0.171	-0.363
	(1.314)	(0.499)	(1.313)	(0.998)	(0.691)	(1.324)
Current Occupational Class (NS-SEC8) *(Ref*: *Inapplicable or no occupation*)						
Large employers & higher management	2.468***	1.663***	1.483***	0.093	-0.167	-0.011
	(5.734)	(4.987)	(3.972)	(0.787)	(1.408)	(0.018)
Higher professional	1.931***	1.455***	1.701***	0.192	-0.019	0.108
	(5.618)	(5.648)	(3.303)	(1.530)	(0.099)	(0.153)
Lower management & professional	1.103***	1.256***	1.002***	0.348*	-0.161	0.258
	(3.814)	(5.856)	(2.966)	(2.037)	(0.772)	(0.410)
Intermediate	0.871***	0.985***	0.486	0.216	-0.271	0.148
	(3.062)	(6.476)	(1.344)	(1.446)	(1.389)	(0.220)
Small employers	0.220	0.560***	0.063	2.507***	1.575***	1.375***
	(0.737)	(3.263)	(0.223)	(5.909)	(4.315)	(3.169)
Lower supervisory & technical	0.772*	1.103***	0.675***	1.750***	1.270***	1.533**
	(1.995)	(4.006)	(2.855)	(4.386)	(4.659)	(2.592)
Semi-routine	0.415	0.982***	0.674*	1.033***	1.174***	1.011***
	(1.626)	(5.437)	(1.774)	(3.469)	(4.669)	(2.822)
Routine	0.272	0.305	0.003	0.764**	0.929***	0.570
	(1.227)	(1.398)	(0.011)	(2.438)	(5.435)	(1.594)
Father’s Education *(Ref*. *Father did not go to School)*						
Father left school with no qualification				0.278	0.439	0.055
				(0.820)	(1.284)	(0.210)
Father some qualification				0.617	0.935**	0.873**
				(1.353)	(2.484)	(2.592)
Father post-school qualification				0.442	0.906***	0.693
				(1.507)	(3.398)	(1.561)
Father university or higher degree				0.213	0.358	-0.174
				(0.860)	(1.089)	(0.463)
Constant	-23.765***	-29.770***	-16.830*	-24.041***	-35.651***	-15.454
	(4.647)	(5.680)	(1.817)	(3.898)	(5.818)	(1.717)
*R*^2^	0.21	0.20	0.19	0.22	0.23	0.20
*N*	5,171	3,746	2,371	3,812	2,616	1,973

Notes:

* p<0.1

** *p*<0.05

*** *p*<0.01.

All specifications include age dummies, region dummies and wave dummies. The columns denoted by (b) also include, as controls, region and wave interactions together with paternal education. Standard errors are clustered at the country of origin level.

In the Supporting Information, we show that differences in culture are also important to explain the propensity to save and we find strong effects, in terms of both magnitude and significance (S4 Table).

One of the potential concerns with Table 1 is that saving rates might suffer from self-reporting bias. To address such a concern, our dataset contains records on individual’s wealth, which allow us to calculate individual’s specific savings from wealth differences across time to match the same estimates as in Table 1. S5 Table reports the estimates of regressing saving rates based on wealth differences from wave 2 to wave 4 on the country of origin savings rates. The right-hand side variables are measured in wave 4. The results are consistent with the other two saving measures. The effect of culture on the change in wealth has a similar impact across the three generations.

## Conclusions

This study examines the effect of culture and its persistence on savings. We show evidence of a robust association between immigrant saving behavior and the saving rates in their country of origin that persists up to the third generation. Our results are consistent across different measures of savings (two self-reported savings measures and savings calculated as wealth change over time). These results go against the prevailing evidence suggesting that culture does not play a role in shaping savings behavior, and instead indicate that culture cannot be disregarded in the study of saving differences across countries.

## Supporting information

S1 Table List of countries of origin and number of observations for different generations of immigrants. (DOCX)

S2 Table Summary statistics. (DOCX)

S3 Table Log amount saved, controlling for permanent income. All specifications include age dummies, region dummies, wave dummies and eight occupational class indicators (as in Table 1). The columns denoted by (b) also include, as controls, region and wave interactions together with paternal education. Standard errors are clustered at the country of origin level.
(DOCX)

S4 Table Propensity to save: Probit estimates for whether an individual saves or not. (DOCX)

S5 Table Positive savings: “Amount of increase in wealth between wave 2 and 4”. *** p<0.01, ** p<0.05, * p<0.1. All specifications include full age and region dummies; the specifications in columns (b) additionally include father’s education as controls. Standard errors are clustered at the country of origin level.
(DOCX)

S1 File Replication package. This zip file contains the STATA do-file used to replicate the results of the manuscript. The Understanding Society Survey data can be obtained from: https://www.understandingsociety.ac.uk.
(ZIP)
