# Supplementary material for: The cultural origin of saving behavior
Source: PLoS One. 2018 Sep 12;13(9):e0202290. doi: 10.1371/journal.pone.0202290 (PMC6135367; doi:10.1371/journal.pone.0202290)
Supplement: S1 Table — (DOCX) [file pone.0202290.s001.docx]

Supporting information

**S1 Table. List of countries of origin and number of observations for different generations of immigrants**

| **Countries of Origin** | **First Generation** | **Second Generation** | **Third Generation** |
| --- | --- | --- | --- |
| Ireland | 261 | 797 | 1,299 |
| France | 75 | 46 | 62 |
| Germany | 218 | 174 | 144 |
| Italy | 84 | 103 | 124 |
| Spain | 43 | 25 | 33 |
| Poland | 264 | 87 | 137 |
| Cyprus | 48 | 47 | 103 |
| Turkey | 66 | 16 | 7 |
| Australia | 67 | 34 | 27 |
| New Zealand | 48 | 16 | 6 |
| Canada | 48 | 83 | 72 |
| US | 120 | 86 | 79 |
| China/HK | 163 | 60 | 12 |
| India | 901 | 596 | 104 |
| Pakistan | 784 | 524 | 9 |
| Bangladesh | 652 | 276 | 3 |
| Sri Lanka | 204 | 35 | 6 |
| Kenya | 168 | 84 | 14 |
| Ghana | 175 | 47 | 5 |
| Nigeria | 253 | 104 | 3 |
| Uganda | 84 | 28 | 1 |
| South Africa | 153 | 61 | 31 |
| Jamaica | 292 | 417 | 90 |
| Total | 5,171 | 3,746 | 2,371 |
