# Supplementary material for: The cultural origin of saving behavior
Source: PLoS One. 2018 Sep 12;13(9):e0202290. doi: 10.1371/journal.pone.0202290 (PMC6135367; doi:10.1371/journal.pone.0202290)
Supplement: S2 Table — (DOCX) [file pone.0202290.s002.docx]

Supporting information

**S2 Table. Summary statistics**

|  |  | **First Generation** | | | **Second Generation** | | | | **Third Generation** | | | |
| --- | --- | --- | --- | --- | --- | --- | --- | --- | --- | --- | --- | --- |
|  |  | **Obs.** | **Mean** | **St. dev** | | **Obs.** | **Mean** | **St. dev** | | **Obs.** | **Mean** | **St. dev** |
| Log Amount Saved | | 5,171 | 1.327 | 2.249 | | 3,746 | 1.832 | 2.451 | | 2,371 | 1.865 | 2.422 |
| Dom Savings/GDP | | 5,171 | 0.200 | 0.075 | | 3,746 | 0.224 | 0.082 | | 2,371 | 0.280 | 0.071 |
| Saves |  | 5,138 | 0.278 | 0.448 | | 3,741 | 0.383 | 0.486 | | 2,363 | 0.398 | 0.490 |
| Positive Savings | | 3,330 | 3.857 | 27.614 | | 2,637 | 4.560 | 62.258 | | 1,843 | 3.709 | 24.621 |
| Female |  | 5,171 | 0.531 | 0.499 | | 3,746 | 0.568 | 0.495 | | 2,371 | 0.577 | 0.494 |
| Married |  | 5,171 | 0.642 | 0.480 | | 3,746 | 0.436 | 0.496 | | 2,371 | 0.461 | 0.499 |
| Number of Children | | 5,171 | 0.975 | 1.222 | | 3,746 | 0.841 | 1.146 | | 2,371 | 0.608 | 0.978 |
| Log Monthly Income | | 5,171 | 9.719 | 0.110 | | 3,746 | 9.727 | 0.117 | | 2,371 | 9.717 | 0.140 |
| College and above | | 5,171 | 0.328 | 0.470 | | 3,746 | 0.299 | 0.458 | | 2,371 | 0.261 | 0.439 |
| Other higher degree | | 5,171 | 0.101 | 0.301 | | 3,746 | 0.125 | 0.331 | | 2,371 | 0.117 | 0.321 |
| A-Level degree | | 5,171 | 0.148 | 0.355 | | 3,746 | 0.238 | 0.426 | | 2,371 | 0.229 | 0.420 |
| Secondary education | | 5,171 | 0.131 | 0.338 | | 3,746 | 0.202 | 0.402 | | 2,371 | 0.188 | 0.391 |
| Unemployed |  | 5,171 | 0.072 | 0.259 | | 3,746 | 0.093 | 0.291 | | 2,371 | 0.057 | 0.233 |
| Out of the labor force | | 5,171 | 0.411 | 0.492 | | 3,746 | 0.333 | 0.471 | | 2,371 | 0.386 | 0.487 |
| Father left school with no qualification | | 3,812 | 0.268 | 0.443 | | 2,616 | 0.381 | 0.486 | | 1,973 | 0.397 | 0.489 |
| Father some qualification | | 3,812 | 0.269 | 0.444 | | 2,616 | 0.253 | 0.435 | | 1,973 | 0.200 | 0.400 |
| Father post-school qualification | | 3,812 | 0.164 | 0.370 | | 2,616 | 0.178 | 0.382 | | 1,973 | 0.278 | 0.448 |
| Father university or higher degree | | 3,812 | 0.193 | 0.395 | | 2,616 | 0.126 | 0.332 | | 1,973 | 0.119 | 0.324 |
| Large employers & higher management | | 5,171 | 0.017 | 0.130 | | 3,746 | 0.029 | 0.167 | | 2,371 | 0.035 | 0.184 |
| Higher professional | | 5,171 | 0.058 | 0.234 | | 3,746 | 0.058 | 0.235 | | 2,371 | 0.050 | 0.218 |
| Lower management & professional | | 5,171 | 0.126 | 0.332 | | 3,746 | 0.180 | 0.384 | | 2,371 | 0.190 | 0.393 |
| Intermediate | | 5,171 | 0.063 | 0.242 | | 3,746 | 0.104 | 0.305 | | 2,371 | 0.079 | 0.270 |
| Small employers | | 5,171 | 0.057 | 0.232 | | 3,746 | 0.053 | 0.224 | | 2,371 | 0.052 | 0.222 |
| Lower supervisory & technical | | 5,171 | 0.032 | 0.176 | | 3,746 | 0.032 | 0.175 | | 2,371 | 0.045 | 0.207 |
| Semi-routine | | 5,171 | 0.123 | 0.328 | | 3,746 | 0.115 | 0.319 | | 2,371 | 0.090 | 0.287 |
| Routine | | 5,171 | 0.070 | 0.255 | | 3,746 | 0.044 | 0.204 | | 2,371 | 0.051 | 0.219 |
