# Supplementary material for: The cultural origin of saving behavior
Source: PLoS One. 2018 Sep 12;13(9):e0202290. doi: 10.1371/journal.pone.0202290 (PMC6135367; doi:10.1371/journal.pone.0202290)
Supplement: S3 Table — All specifications include age dummies, region dummies, wave dummies and eight occupational class indicators (as in Table 1). The columns denoted by (b) also include, as controls, region and wave interactions together with paternal education. Standard errors are clustered at the country of origin level. (DOCX) [file pone.0202290.s003.docx]

Supporting information

**S3 Table. Log Amount Saved, controlling for permanent income**

| Variables | 1st Gen | 2nd Gen | 3rd Gen | 1st Gen (b) | 2nd Gen (b) | 3rd Gen (b) |
| --- | --- | --- | --- | --- | --- | --- |
| Dom. savings/GDP | 1.508** | 1.158** | 0.847* | 1.617** | 1.608** | 0.759 |
|  | (2.729) | (2.255) | (1.803) | (2.721) | (2.812) | (1.559) |
| Female | 0.012 | -0.053 | -0.102 | -0.002 | -0.061 | -0.082 |
|  | (0.154) | (0.962) | (1.261) | (0.023) | (0.928) | (1.277) |
| Married | -0.301 | -0.271 | -1.025* | -0.080 | -0.667 | -0.832 |
|  | (0.717) | (0.596) | (1.949) | (0.186) | (1.099) | (1.260) |
| Number of children | -0.169* | -0.231** | -0.541*** | -0.129 | -0.348*** | -0.539*** |
|  | (1.737) | (2.654) | (4.747) | (1.380) | (3.502) | (3.440) |
| Permanent income1 | 2.948 | 5.570 | 27.123** | -1.530 | 13.858 | 22.868 |
|  | (0.361) | (0.630) | (2.119) | (0.181) | (1.109) | (1.469) |
| Log Monthly Income | 2.566*** | 3.102*** | 1.774* | 2.613*** | 3.740*** | 1.600 |
|  | (4.738) | (5.995) | (1.882) | (4.053) | (6.654) | (1.705) |
| Education *(Ref. No Qualification)* | | |  |  |  |  |
| College and above | 0.379 | 0.532 | -0.416 | 0.600* | 0.366 | -0.114 |
|  | (1.180) | (1.636) | (0.708) | (1.829) | (0.760) | (0.164) |
| Secondary education, | 0.193 | 0.069 | -0.032 | 0.316** | 0.055 | 0.114 |
| A-level and other higher degree | (1.468) | (0.484) | (0.112) | (2.400) | (0.228) | (0.327) |
|  |  |  |  |  |  |  |
| Employment Status *(Ref: Employed)* | | |  |  |  |  |
| Unemployed | -0.476* | -0.345* | -0.766** | -0.491* | -0.452* | -0.802** |
|  | (2.053) | (1.991) | (2.337) | (1.873) | (1.961) | (2.258) |
| Out of Labor Force | -0.307 | -0.093 | -0.350 | -0.244 | -0.174 | -0.367 |
|  | (1.317) | (0.489) | (1.302) | (0.998) | (0.714) | (1.315) |
| Father’s Education *(Ref. Father did not go to School)* | | | |  |  |  |
| Father left school with no qualification | | |  | 0.113 | -0.175 | -0.008 |
|  |  |  |  | (0.856) | (1.489) | (0.014) |
| Father some qualification |  |  |  | 0.213 | -0.012 | 0.114 |
|  |  |  |  | (1.534) | (0.063) | (0.163) |
| Father post-school qualification |  |  |  | 0.371* | -0.157 | 0.265 |
|  |  |  |  | (1.987) | (0.764) | (0.430) |
| Father university or higher degree |  |  |  | 0.240 | -0.280 | 0.157 |
|  |  |  |  | (1.506) | (1.447) | (0.236) |
| Constant | -52.505 | -83.710 | -279.839** | -9.439 | -169.754 | -237.154 |
|  | (0.658) | (0.968) | (2.326) | (0.111) | (1.411) | (1.625) |
| *R*^2^ | 0.21 | 0.20 | 0.19 | 0.21 | 0.22 | 0.20 |
| *N* | 5,170 | 3,746 | 2,371 | 3,811 | 2,616 | 1,973 |

All specifications include age dummies, region dummies, wave dummies and eight occupational class indicators (as in Table 1). The columns denoted by (b) also include, as controls, region and wave interactions together with paternal education. Standard errors are clustered at the country of origin level.
