# Supplementary material for: The cultural origin of saving behavior
Source: PLoS One. 2018 Sep 12;13(9):e0202290. doi: 10.1371/journal.pone.0202290 (PMC6135367; doi:10.1371/journal.pone.0202290)
Supplement: S4 Table — (DOCX) [file pone.0202290.s004.docx]

Supporting information

**S4 Table. Propensity to save: Probit estimates for whether an individual saves or not**

| VARIABLES | 1^st^ Gen | 2^nd^ Gen | 3^rd^ Gen | 1^st^ Gen (b) | 2^nd^ Gen (b) | 3^rd^ Gen (b) |
| --- | --- | --- | --- | --- | --- | --- |
|  |  |  |  |  |  |  |
| Dom. savings/GDP | 0.295** | 0.274** | 0.236** | 0.326** | 0.307* | 0.220** |
|  | -0.123 | (0.126) | (0.120) | (0.130) | (0.159) | (0.109) |
| Female | 0.027 | 0.004 | -0.002 | 0.027 | 0.000 | -0.004 |
|  | (0.017) | (0.012) | (0.020) | (0.021) | (0.016) | (0.021) |
| Married | -0.022 | 0.015 | 0.087*** | -0.014 | 0.016 | 0.082*** |
|  | (0.019) | (0.021) | (0.015) | (0.021) | (0.020) | (0.017) |
| Number of children | -0.025 | -0.028** | -0.063*** | -0.024* | -0.037*** | -0.077*** |
|  | (0.015) | (0.011) | (0.014) | (0.014) | (0.010) | (0.010) |
| Education *(Ref. No Qualification)* | | |  |  |  |  |
| College and above | 0.148*** | 0.171*** | 0.171*** | 0.146*** | 0.214*** | 0.205*** |
|  | (0.035) | (0.028) | (0.038) | (0.036) | (0.036) | (0.031) |
| Other higher degree | 0.116** | 0.056 | 0.162*** | 0.116*** | 0.111** | 0.197*** |
|  | (0.047) | (0.047) | (0.039) | (0.041) | (0.047) | (0.033) |
| A level degree | 0.110*** | 0.043 | 0.142*** | 0.117*** | 0.050 | 0.146*** |
|  | (0.031) | (0.033) | (0.031) | (0.029) | (0.042) | (0.030) |
| Secondary Education | 0.087*** | 0.019 | 0.080** | 0.105*** | 0.032 | 0.108*** |
|  | (0.026) | (0.030) | (0.034) | (0.026) | (0.039) | (0.040) |
| Employment Status *(Ref: Employed)* | |  |  |  |  |  |
| Unemployed | -0.135*** | -0.125** | -0.182*** | -0.154*** | -0.139** | -0.189** |
|  | (0.041) | (0.049) | (0.059) | (0.049) | (0.063) | (0.076) |
| Out of Labour Force | -0.045 | -0.003 | -0.003 | -0.031 | -0.019 | -0.006 |
|  | (0.051) | (0.049) | (0.038) | (0.053) | (0.052) | (0.049) |
| Father’s education level *(Ref: Father did not go to school)* | | | | | | |
| Father university or higher degree | |  |  | 0.058 | -0.052* | 0.043 |
|  |  |  |  | (0.037) | (0.030) | (0.139) |
| Father post-school qualification | |  |  | 0.079** | -0.012 | 0.078 |
|  |  |  |  | (0.034) | (0.050) | (0.158) |
| Father some qualification | |  |  | 0.109*** | -0.034 | 0.100 |
|  |  |  |  | (0.039) | (0.049) | (0.143) |
| Father left school with no qualification | |  |  | 0.085** | -0.071* | 0.103 |
|  |  |  |  | (0.037) | (0.037) | (0.150) |
| Current Occupational Class (NS-SEC8) *(Ref: Inapplicable or no occupation*) | | | | | | |
| Large employers & higher management | 0.469*** | 0.319*** | 0.333*** | 0.484*** | 0.336*** | 0.297*** |
|  | (0.069) | (0.064) | (0.047) | (0.071) | (0.054) | (0.068) |
| Higher professional | 0.433*** | 0.304*** | 0.362*** | 0.414*** | 0.307*** | 0.325*** |
|  | (0.064) | (0.052) | (0.058) | (0.074) | (0.054) | (0.079) |
| Lower management & professional | 0.295*** | 0.289*** | 0.273*** | 0.283*** | 0.302*** | 0.267*** |
|  | (0.061) | (0.045) | (0.051) | (0.067) | (0.044) | (0.064) |
| Intermediate | 0.245*** | 0.237*** | 0.194*** | 0.235*** | 0.268*** | 0.204*** |
|  | (0.066) | (0.033) | (0.052) | (0.073) | (0.037) | (0.062) |
| Small employers | 0.102 | 0.140*** | 0.073 | 0.109 | 0.148** | 0.060 |
|  | (0.064) | (0.046) | (0.047) | (0.073) | (0.071) | (0.054) |
| Lower supervisory & technical | 0.238*** | 0.269*** | 0.201*** | 0.226** | 0.270*** | 0.231*** |
|  | (0.079) | (0.055) | (0.048) | (0.099) | (0.065) | (0.064) |
| Semi-routine | 0.162*** | 0.249*** | 0.219*** | 0.167** | 0.266*** | 0.219** |
|  | (0.061) | (0.039) | (0.067) | (0.071) | (0.049) | (0.093) |
| Routine & Manual Occupations | 0.125** | 0.123* | 0.092** | 0.122** | 0.153*** | 0.020 |
|  | (0.052) | (0.063) | (0.043) | (0.062) | (0.053) | (0.074) |
| Observations | 5,339 | 3,906 | 2,453 | 3,928 | 2,718 | 2,034 |
| Robust standard errors in parentheses * p<0.1; ** p<0.05; *** p<0.01. Probit Estimates – Marginal Effects are reported above. All specifications include full age dummies, 11 region dummies and 2 wave dummies. The specifications in columns (b) additionally include father’s education and region and wave interactions as controls. Standard errors are clustered at the country of origin level. | | | | | | |
