# Supplementary material for: The cultural origin of saving behavior
Source: PLoS One. 2018 Sep 12;13(9):e0202290. doi: 10.1371/journal.pone.0202290 (PMC6135367; doi:10.1371/journal.pone.0202290)
Supplement: S5 Table — *** p<0.01, ** p<0.05, * p<0.1. All specifications include full age and region dummies; the specifications in columns (b) additionally include father’s education as controls. Standard errors are clustered at the country of origin level. (DOCX) [file pone.0202290.s005.docx]

Supporting information

**S5 Table. Positive Savings**

**Table S5:'Positive Savings”**

**“Amount of increase in wealth between Wave 2 and 4”**

| VARIABLES | 1st Gen | 2nd Gen | 3rd Gen | 1st Gen (b) | 2nd Gen (b) | 3rd Gen (b) |
| --- | --- | --- | --- | --- | --- | --- |
|  |  |  |  |  |  |  |
| Dom. savings/GDP | 12.538* | 27.430** | 16.742** | 6.459* | 36.240*** | 13.352** |
|  | (6.723) | (9.817) | (6.354) | (3.676) | (12.588) | (5.708) |
| Female | 0.953 | 0.864 | -0.391 | 0.171 | 1.382 | 0.294 |
|  | (1.071) | (1.448) | (0.479) | (0.961) | (1.711) | (0.437) |
| Married | -3.142 | -1.818 | 3.583** | -0.963 | -2.588 | 3.549*** |
|  | (2.329) | (2.535) | (1.463) | (1.397) | (3.607) | (1.143) |
| Number of children | -0.040 | 0.960 | 0.038 | 0.003 | 0.979 | -0.080 |
|  | (0.377) | (1.512) | (0.289) | (0.460) | (1.783) | (0.284) |
| Log Monthly Income | 24.107* | 93.872 | 3.487* | 14.100** | 120.856 | 2.002 |
|  | (12.106) | (65.055) | (1.971) | (5.792) | (85.725) | (2.533) |
| Education *(Ref. No Qualification)* | | |  |  |  |  |
| College and above | 1.948 | 1.797 | -0.328 | 2.240 | 1.179 | 2.306*** |
|  | (1.325) | (4.252) | (1.385) | (1.586) | (4.801) | (0.682) |
| Other higher degree | -0.847 | 10.495 | -1.902 | -0.278 | 12.445 | 0.488 |
|  | (1.188) | (7.532) | (1.684) | (1.486) | (9.345) | (0.943) |
| A level degree | 0.546 | 1.140 | -1.434* | 0.674 | 0.494 | 1.344*** |
|  | (1.039) | (1.512) | (0.798) | (1.134) | (1.857) | (0.449) |
| Secondary Education | 0.675 | 1.584 | -1.833* | 0.646 | 0.797 | 0.192 |
|  | (1.051) | (1.380) | (1.000) | (1.140) | (1.486) | (0.393) |
| Employment Status *(Ref: Employed)* | |  |  |  |  |  |
| Unemployed | 0.228 | -1.737 | -1.507 | 1.428 | -4.377** | -1.273 |
|  | (2.917) | (1.290) | (0.974) | (3.601) | (1.589) | (0.821) |
| Out of Labour Force | -3.352* | 2.970 | -1.137 | -2.433** | 2.341 | -1.634* |
|  | (1.936) | (3.986) | (0.992) | (1.122) | (4.956) | (0.910) |
| Father’s education level *(Ref: Father did not go to school)* | | | | | | |
| Father university or higher degree | |  |  | 0.770 | 2.447 | 1.131 |
|  |  |  |  | (0.871) | (2.159) | (1.246) |
| Father post-school qualification | |  |  | 0.478 | 3.659 | -1.726 |
|  |  |  |  | (1.425) | (3.195) | (2.200) |
| Father some qualification | |  |  | 0.273 | 10.682 | -0.608 |
|  |  |  |  | (0.844) | (8.388) | (1.664) |
| Father left school with no qualification | |  |  | -0.025 | 1.427 | 0.049 |
|  |  |  |  | (1.118) | (2.271) | (0.953) |
| Current Occupational Class (NS-SEC8) *(Ref: Inapplicable or no occupation*) | | | | | | |
| Large employers & higher management | -1.623 | -16.887* | 4.752 | 1.684 | -23.844* | 5.483 |
|  | (5.175) | (8.801) | (2.787) | (3.738) | (12.797) | (3.579) |
| Higher professional | 1.576 | -16.933* | 2.776 | -2.135 | -23.729* | 2.471 |
|  | (4.284) | (9.736) | (1.926) | (2.986) | (13.493) | (1.934) |
| Lower management & professional | -4.061 | -13.004 | -0.868 | -2.641 | -19.433 | -0.987 |
|  | (3.270) | (8.668) | (1.123) | (2.498) | (12.236) | (1.197) |
| Intermediate | -4.473 | -10.434* | 2.930 | -3.045 | -16.048* | 3.420 |
|  | (2.817) | (5.605) | (2.096) | (2.109) | (8.287) | (3.123) |
| Small employers | -3.434 | -10.054* | -0.873 | -2.693 | -16.677* | -1.666** |
|  | (2.386) | (5.129) | (0.876) | (2.247) | (8.212) | (0.597) |
| Lower supervisory & technical | -4.640* | -13.080** | -0.817 | -5.047** | -20.778** | 0.353 |
|  | (2.693) | (5.835) | (1.685) | (2.101) | (9.754) | (1.498) |
| Semi-routine | -3.788* | -9.644* | -0.222 | -3.306 | -14.048** | -1.183 |
|  | (2.167) | (4.889) | (0.975) | (2.111) | (6.729) | (0.895) |
| Routine & Manual Occupations | -4.505** | -7.486** | 4.593 | -3.814** | -10.772** | -2.835** |
|  | (2.023) | (3.292) | (3.296) | (1.678) | (4.963) | (1.090) |
| Constant | -234.190* | -919.014 | -39.780** | -136.508** | -1,183.052 | -21.599 |
|  | (117.418) | (633.392) | (18.890) | (56.004) | (837.081) | (23.551) |
| Observations | 3,295 | 2,604 | 1,831 | 2,657 | 1,991 | 1,566 |
| R-squared | 0.035 | 0.054 | 0.064 | 0.039 | 0.068 | 0.069 |

*** p<0.01, ** p<0.05, * p<0.1. All specifications include full age and region dummies; the specifications in columns (b) additionally include father’s education as controls. Standard errors are clustered at the country of origin level.
